# Stem-cell regulation in periodontium formation and regeneration

**DOI:** 10.1007/s00774-026-01724-0

**Published:** 2026-04-22

**Authors:** Mizuki Nagata, Chika Nagaoka, Takanori Iwata

**Affiliations:** Department of Periodontology, Graduate School of Medical and Dental Sciences, Institute of Science Tokyo, Tokyo, Japan

**Keywords:** Mesenchymal progenitor cells, Periodontium, Dental follicle, Apical papilla, In vivo lineage-tracing experiments, Mouse genetic models

## Abstract

**Introduction:**

The periodontium is a dynamic composite tissue that anchors teeth to the alveolar bone and adapts continuously to mechanical loading and inflammatory challenges throughout life. Its development, homeostasis, and regeneration depend on diverse mesenchymal stem and progenitor cell populations derived from the dental follicle, apical papilla, and periodontal ligament.

**Results:**

Recent advances in in vivo lineage-tracing studies have demonstrated that these progenitors are heterogeneous, and lineage restricted, rather than constituting a uniform stem cell pool. Distinct populations, including PTHrP⁺ dental follicle–derived progenitors and CXCL12⁺ apical papilla cells, contribute differentially to cementum, periodontal ligament, and alveolar bone formation during tooth root development and postnatal remodeling. The fate decisions of these progenitors are tightly regulated by coordinated signaling pathways, including Hedgehog–Foxf, Wnt/β-catenin, and BMP/TGF-β signaling, as well as by mechanotransducive cues. Spatially and temporally precise regulation of these pathways is essential for proper cementogenesis and alveolar bone formation, whereas their dysregulation leads to impaired periodontal regeneration and bone loss.

**Summary:**

This review summarizes current knowledge of periodontal stem cell biology with a particular emphasis on developmental origins and in vivo regulatory mechanisms and discusses emerging concepts that support endogenous stem cell–based approaches for periodontal regeneration and future therapeutic strategies.

## Introduction

The periodontium is a highly specialized composite tissue comprising the gingiva, periodontal ligament (PDL), alveolar bone, and the cementum covering the root surface. It anchors the teeth to the alveolar bone and facilitates the transfer of occlusal forces while maintaining continuous remodeling throughout life. The periodontium primarily functions to provide structural support for the teeth, perceive mechanical stimuli associated with mastication and speech, and serve as a defensive barrier against bacterial invasion, thereby maintaining oral tissue homeostasis [1, 2]. Maintenance of this complex interface depends on a resident pool of stem and progenitor cells capable of self-renewal and multilineage differentiation. Indeed, a mesenchymal stem-cell population in the PDL, called periodontal ligament stem cells (PDLSCs), has been isolated and applied for regenerative therapies [3–6].

The periodontium develops in concert with tooth root formation through reciprocal and sequential interactions between the dental mesenchyme and epithelium [7–9]. Developmentally, the periodontium—particularly the attachment apparatus, including the cementum, periodontal ligament (PDL), and alveolar bone—originates from the dental follicle (DF), a sac-like mesenchymal tissue enveloping the developing tooth germ [2, 8]. Nevertheless, how these stem cells make a cell fate decision into the attachment apparatus remains largely unresolved. To achieve a better regenerative process, in vivo stem-cell regulation and differentiation need to be investigated. Recently, we identified two distinct stem-cell populations that contribute to alveolar bone formation and to tooth root, including cementum [10, 11].

In this review, we summarize current knowledge of stem-cell populations within the periodontium, and how these stem cells contribute to periodontal tissue development, homeostasis, and repair, with a particular focus on insights derived from mouse lineage-tracing studies.

## Developmental process of the periodontium

During early embryogenesis, neural crest cells (NCCs) emerge from the neural tube and migrate into the branchial arches, where they contribute to the formation of craniofacial skeletal and neural tissues [12, 13]. The proto-oncogene Wnt family member 1 (*Wnt1*) is transiently expressed in neural crest cells as they delaminate from the neural tube during early embryogenesis. Lineage-tracing studies using *Wnt1*-cre have demonstrated that all dental mesenchymal tissues, including the periodontium, originate from NCCs [14]. Following crown formation, tooth root development is initiated by the elongation of Hertwig’s epithelial root sheath (HERS), which serves not only as a structural guide for root morphogenesis but also as a signaling center that regulates the behavior of surrounding mesenchymal cells. Neural crest-derived mesenchymal cells in the apical region interact with HERS, thereby coordinating tooth root development and formation of the surrounding periodontal tissues. The DF, a mesenchymal sac enveloping the developing tooth germ, constitutes the primary reservoir of progenitor cells that give rise to the cementum, PDL, and alveolar bone [2, 8].

Parathyroid hormone-related protein (PTHrP), a locally acting autocrine and paracrine factor, plays a pivotal role in regulating epithelial–mesenchymal interactions across multiple organs, including the developing tooth [15]. In endochondral bone development, PTHrP-expressing chondrocytes within the resting zone of the postnatal growth plate function as skeletal stem cells [16], indicating that PTHrP-expressing mesenchymal cells exhibit characteristics of skeletal stem or progenitor populations. During tooth development, PTHrP is expressed in the dental follicle (DF) and is essential for regulating tooth eruption through the promotion of osteoclastogenesis [17–19]. Among DF-derived populations, PTHrP-expressing cells function as mesenchymal progenitors that differentiate into PDL fibroblasts, cementoblasts, and alveolar osteoblasts through autocrine PTHrP–PPR signaling, thereby regulating the fate of DF-derived progenitors [19, 20].

Recent lineage-tracing studies have further identified multiple DF subpopulations marked by *glioma-associated oncogene homolog 1 (Gli1)-creER* or *Osx-creER*, all of which contribute to periodontal tissue formation [18, 21, 22]. *Gli-creER* marks a broad range of dental epithelial and mesenchymal cells in DP and DF, whereas *Osx-creER* marks mesenchymal cells surrunding HERS. In contrast, *PTHrP-creER*^+^ cells were explicitly localized to the inner layer of DF adjacent to HERS, representing a highly Hedgehog-responsive subset of Gli1^+^ cells [11].

Collectively, these findings highlight that the DF harbors heterogeneous mesenchymal progenitor cell populations that orchestrate the development and regeneration of periodontal tissues.

## Two distinct origins of cementum on the root surface

Cementum is a unique mineralized tissue covering the tooth root surface and forms a critical interface between the tooth and the PDL [23]. Functionally, cementum is indispensable for maintaining periodontal attachment and preventing ankylosis, thereby enabling efficient dissipation of masticatory forces. Cementum is traditionally categorized into acellular and cellular types based on the presence or absence of embedded cementocytes [24]. Acellular cementum forms a thin mineralized layer covering the cervical portion of the tooth root, whereas cellular cementum constitutes a thicker layer localized to the apical region. Structurally, cementum comprises two types of fibers: extrinsic (Sharpey’s) fibers, which represent the embedded termini of principal periodontal fibers, and intrinsic fibers produced by cementoblasts that form the cementum matrix itself [23, 25]. Successful periodontal regeneration is considered to rely on the formation of functionally competent acellular cementum that is strongly connected to alveolar bone via the PDL and Sharpey’s fiber [26, 27].

Cementogenesis originates from neural crest-derived ectomesenchymal cells during tooth root formation. The DF has traditionally been considered as the principal source of cementoblasts [2, 24, 28], consistent with our lineage-tracing analysis demonstrating that PTHrP^+^ DF cells differentiate into cementoblasts contributing specifically to acellular cementum [19]. Chemokine C–X–C motif ligand 12 (CXCL12), also known as stromal cell-derived factor 1a (SDF-1a), is a potent *α*-chemokine that attracts mesenchymal stem cells (MSCs) and endothelial progenitor cells via its receptor CXCR4 [29]. In adult tissues, repair and regeneration following injury involve the selective recruitment of circulating or resident stem-cell populations [30, 31]. Dental papilla cells and stem cells from the apical papilla (SCAPs) cells produce abundant chemokines and cytokines, including CXCL9, CXCL12, and CCL2 [32–35]. In the bone marrow, CXCL12 is secreted by specialized reticular stromal cells known as CXCL12-abundant reticular (CAR) cells, which serve as precursors for osteoblasts and marrow adipocytes [36]. Thus, CXCL12 functions as a key chemokine that orchestrates stem-cell migration and coordinates tissue repair processes.

**Fig. 1:**
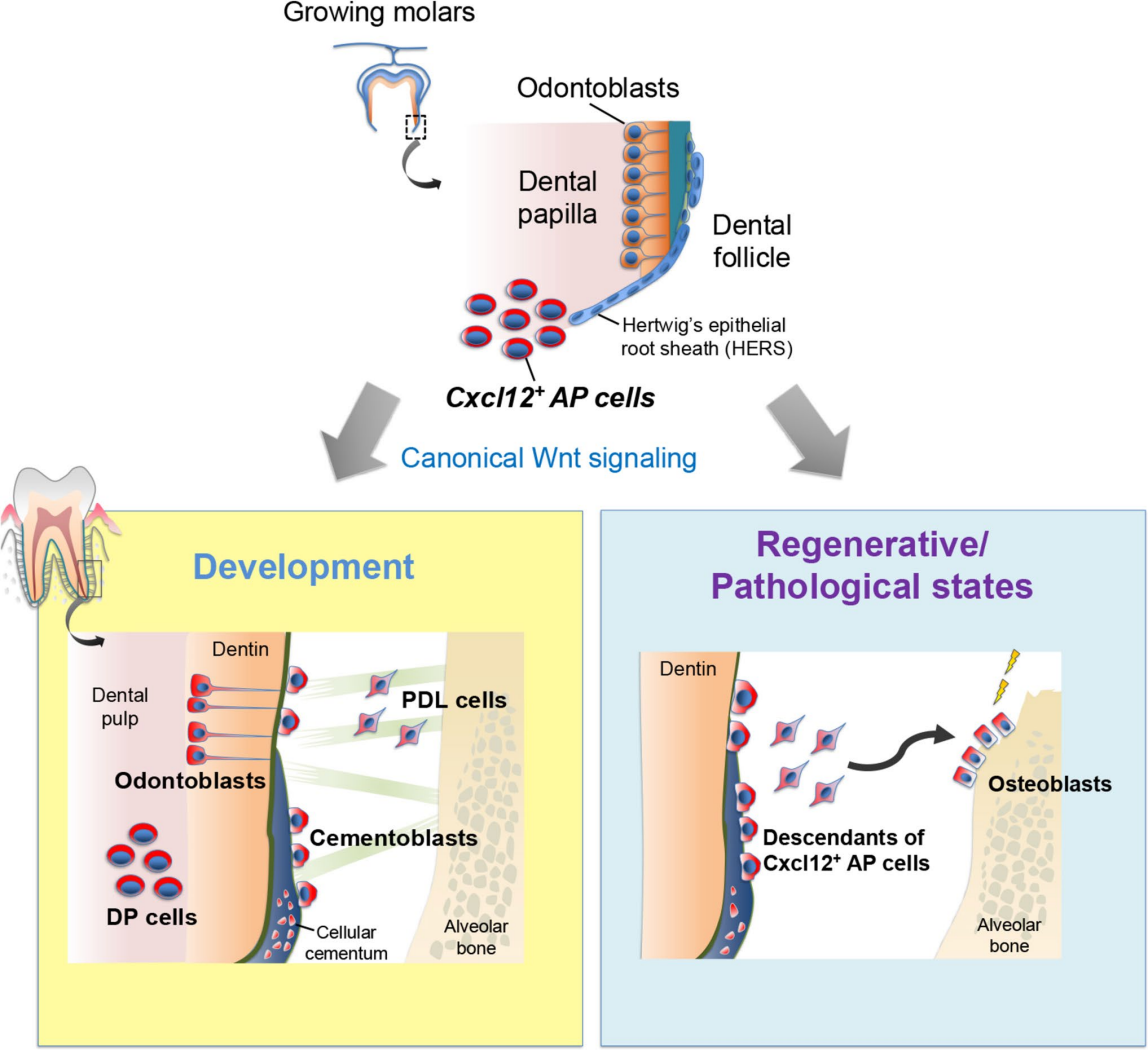
Lineage-tracing of CXCL12^+^ AP cells during development and under regenerative/pathological conditions. Schematic diagrams of CXCL12 AP^+^ cell fates. CXCL12-expressing mesenchymal progenitors in the apical papilla expand along the developing root surface, where their progeny differentiate into odontoblasts and cementoblasts under physiological conditions via canonical Wnt signaling. Under regenerative or pathological conditions, CXCL12⁺ AP-derived cells additionally give rise to osteoblasts contributing to alveolar bone formation. These findings indicate that CXCL12⁺ AP cells function as multipotent mesenchymal progenitors supporting cementogenesis and osteogenesis in both homeostatic and regenerative contexts

Using *Cxcl12-creER* lineage-tracing, the study demonstrated that these cells populate the dental mesenchyme surrounding HERS and differentiate into both odontoblasts forming root dentin and cementoblasts forming cementum along the entire root surface [10]. Notably, CXCL12⁺ apical papilla (AP) cells continue to provide cementoblasts contributing to cellular cementum after root formation, whereas PTHrP^+^ DF cells differentiate into cementoblasts on acellular cementum only during tooth root development. Wnt signaling is the most prominent pathway to contribute to cementogenesis [37]. At the molecular level, canonical Wnt/β-catenin signaling is indispensable for maintaining the odontogenic and cementogenic fates of CXCL12⁺ AP cells. Loss of β-catenin in these cells (*Cxcl12-creER; Ctnnb1*^*fl/fl*^) results in truncated roots with defective dentin and cementum and aberrant differentiation into fibroblast- or chondrocyte-like cells. Wnt ligands derived from HERS epithelial cells act on AP cells ensuring proper odonto-cementogenic differentiation. Inhibition of downstream TGF-β signaling can partially rescue these aberrant fates, indicating cross-talk between Wnt and TGF-β pathways in cementoblast differentiation [10] (Fig. 1).

## Cell fate decisions toward alveolar bone osteoblasts

Alveolar bone development is a tightly regulated process intimately linked to tooth root formation. Alveolar bone forms through intramembranous ossification and originates from the cranial neural crest-derived dental mesenchyme within the DF surrounding the developing tooth germ [38]. Tooth germs undergo continuous positional adjustment within the alveolar bone during jaw growth, inducing bone remodeling through coordinated resorption and formation. Like bone at other sites, alveolar bone provides mechanical support, houses bone marrow, and serves as a reservoir for calcium ions.

We have demonstrated that a key regulatory mechanism underlying this process is the transient activation followed by suppression of the Hedgehog signaling pathway and its downstream effector Foxf1 in a subpopulation of PTHrP^+^ DF cells [11]. These cells are initially Hedgehog responsive, maintaining an undifferentiated state; however, as root formation progresses, Hedgehog and Foxf1 expression are downregulated, permitting commitment toward alveolar osteoblast and PDL lineages. Constitutive activation of Hedgehog in PTHrP⁺ DF cells suppresses alveolar bone formation and results in bone loss. Thus, precise temporal inactivation of the Hedgehog–Foxf axis is essential for differentiation of DF-derived progenitors into alveolar bone osteoblasts and formation of the supporting bone structure. This finding introduces a tooth-specific paradigm in which dynamic on–off regulation of the signaling pathway governs coordinated root and alveolar bone development.

## Diverse stem-cell population in periodontal ligament

The PDL is a specialized connective tissue located between the cementum covering the tooth root and the alveolar bone of the socket. Histologically, it is a thin, fibrous, tendon-like tissue rich in type I collagen fibers (Sharpey’s fibers) that insert into cementum and alveolar bone. The PDL contains a heterogeneous population of fibroblasts, cementoblasts, osteoblasts, endothelial cells, nerve fibers, and perivascular cells [8]. Functionally, the PDL provides mechanical support and shock absorption by anchoring the tooth to alveolar bone and transmitting occlusal forces. Importantly, it also serves as a reservoir of mesenchymal stem/progenitor cells capable of differentiating into cementoblasts, osteoblasts, and fibroblasts, thereby forming the cellular basis for periodontal tissue regeneration [39]. Seo et al. first identified periodontal ligament stem cells (PDLSCs) from human PDL [3]. These clonogenic, multipotent cells exhibited osteogenic, adipogenic, and chondrogenic differentiation in vitro and form cementum/PDL-like structures in vivo. Since this discovery, PDLSC-based regenerative therapies have been widely explored [40].

Recent in vivo lineage-tracing studies have revealed that multiple mesenchymal progenitor populations within the PDL collectively maintain periodontal homeostasis and regeneration. Distinct lineages characterized by the expression of *Gli1*, *Axin2*, and leptin receptor (*LepR*)—have been functionally defined as contributors to PDL remodeling, cementogenesis, and alveolar bone maintenance.

*Gli1*, a transcription factor and a nuclear effector of Hedgehog signaling, marks mesenchymal stem-cell (MSC)-like populations in various tissues, including long bones, calvarial sutures, and the incisor mesenchyme in mice [41–44]. Within the periodontium, GLI1⁺ mesenchymal cells are located near the neurovascular bundle and apical region after root formation, where they function as in vivo PDLSCs that contribute to injury repair and tissue regeneration [45]. Functional disruption of Wnt signaling in GLI1⁺ PDLSCs results in alveolar bone loss, and the Wnt inhibitor sclerostin, secreted by osteocytes, negatively regulates Gli1⁺ cell activity. Notably, occlusal unloading suppresses Wnt signaling and inhibits GLI1⁺ cell activation, suggesting that mechanical forces indirectly modulate GLI1⁺ PDLSCs through Wnt-dependent pathways. Consistent with this, the ablation of GLI1⁺ cells leads to impaired bone remodeling during orthodontic tooth movement, associated with defective mechanotransduction mediated by Yes-associated protein (Yap) [46]. In addition, GLI1⁺ cells contribute to cementum formation via Wnt signaling; constitutive activation of β-catenin within these cells increases cementum thickness, whereas its conditional deletion reduces it [47]. Beyond the PDL, GLI1⁺ cells have also been identified in the alveolar bone marrow, where they participate in bone remodeling and dental implant osseointegration [48]. Collectively, these findings highlight the role of GLI1⁺ mesenchymal progenitors as a key population of in vivo PDLSCs that maintain periodontal and alveolar bone homeostasis through coordinated Hedgehog–Wnt–mechanical signaling cross-talk.

*Axin2*, a direct transcriptional target of the canonical Wnt/β-catenin pathway [49, 50], marks Wnt-responsive mesenchymal progenitors in diverse tissues, including the dental pulp of mouse incisors and molars [51–55]. Within the periodontal ligament (PDL), AXIN2⁺ cells localize predominantly along alveolar bone and cementum surfaces and are situated adjacent to blood vessels, consistent with a perivascular niche of Wnt-responsive progenitors that contribute to periodontal hard tissue dynamics [56, 57]. Lineage-tracing studies demonstrate that AXIN2⁺ PDL cells differentiate into alveolar bone osteoblasts during extraction socket healing and in response to orthodontic tension, where their numbers increase in a Wnt/β-catenin-synchronized manner and their ablation markedly reduces new bone formation and mineral apposition rates, highlighting their mechanosensitive osteogenic function in vivo [57, 58]. In cementogenesis, AXIN2⁺ mesenchymal PDL cells are key progenitors that produce both cellular and acellular cementum, with loss of these cells resulting in hypoplastic cementum and activation of Wnt/β-catenin signaling in AXIN2⁺ lineages leading to cementum overgrowth and hyperplasia, indicating that Wnt activity modulates their cementogenic output [59, 60]. Beyond Wnt, BMP signaling via BMPR1A has emerged as a critical regulator of AXIN2⁺ PDL progenitors during periodontium development: conditional deletion of Bmpr1a in AXIN2-lineage cells causes periodontal defects characterized by alveolar bone loss, impaired cementogenesis, and abnormal Sharpey’s fibers, underscoring the integration of Wnt and BMP pathways in controlling progenitor function [57]. Collectively, these studies position AXIN2⁺ PDL cells as a principal in vivo PDLSC population that integrates canonical Wnt and BMP signaling to regulate alveolar bone remodeling, cementum formation, and mechanotransduction, with perturbed signaling yielding compromised periodontal homeostasis and regeneration.

LEPR⁺ cells represent a distinct population of mesenchymal progenitors that contribute to both skeletal and periodontal homeostasis [61–63]. Within the periodontium, LEPR ⁺ cells are localized primarily around blood vessels in the apical region of the periodontal ligament (PDL) and alveolar bone, where they give rise to osteoblasts, fibroblasts, and, to a lesser extent, cementoblasts during postnatal remodeling [64–66]. Lineage-tracing and ablation studies have demonstrated that LEPR ⁺ cells are indispensable for alveolar bone turnover and maintenance of periodontal integrity, as their depletion results in reduced bone mass and mineral density [66]. Under mechanical stress, LEPR ⁺ PDL cells respond dynamically through Piezo1-mediated mechanotransduction, coordinating cementum and alveolar bone remodeling [67]. During inflammatory challenge, such as periodontitis, LEPR ⁺ cells undergo a fate shift from osteogenic to fibrogenic differentiation, characterized by downregulation of *Osx* and upregulation of Periostin, leading to compromised bone formation [66, 68]. Mechanistically, this functional alteration is associated with suppressed canonical Wnt signaling via the CCRL2–SFRP1 axis, which amplifies inhibition of Wnt3a–β-catenin signaling [68]. Restoration of Wnt activity, either through Ccrl2 deletion or sclerostin neutralization, reinstates the osteogenic potential of LEPR ⁺ cells and promotes alveolar bone regeneration following periodontitis [66]. Moreover, orthodontic force induces periodontal ligament cell apoptosis, which activates LEPR⁺ osteoprogenitors via apoptotic vesicles to drive alveolar bone formation during orthodontic tooth movement [69].

Collectively, these lineage-defined mesenchymal progenitor populations exhibit coordinated spatial and temporal organization rather than functioning as independent entities. During early periodontal development, PTHrP⁺ DF cells and CXCL12⁺ AP cells occupy distinct anatomical niches adjacent to HERS, where they contribute to acellular and cellular cementum formation, respectively, indicating an early functional segregation along the root axis. As development progresses and the periodontium matures, Gli1⁺ cells represent a broader upstream progenitor population that partially overlaps with PTHrP⁺ and other subsets, such as Osx^+^ mesenchymal cells, suggesting a hierarchical relationship in which Gli1⁺ cells give rise to more lineage-restricted progenitors. In the postnatal periodontal ligament, Axin2⁺ and LepR⁺ populations are preferentially localized to perivascular and hard-tissue-adjacent niches, where they contribute to bone remodeling and cementogenesis in a context-dependent manner. Importantly, these populations are not strictly lineage-isolated; instead, they exhibit functional plasticity and partial overlap in response to mechanical loading, injury, or inflammation.

**Fig. 2:**
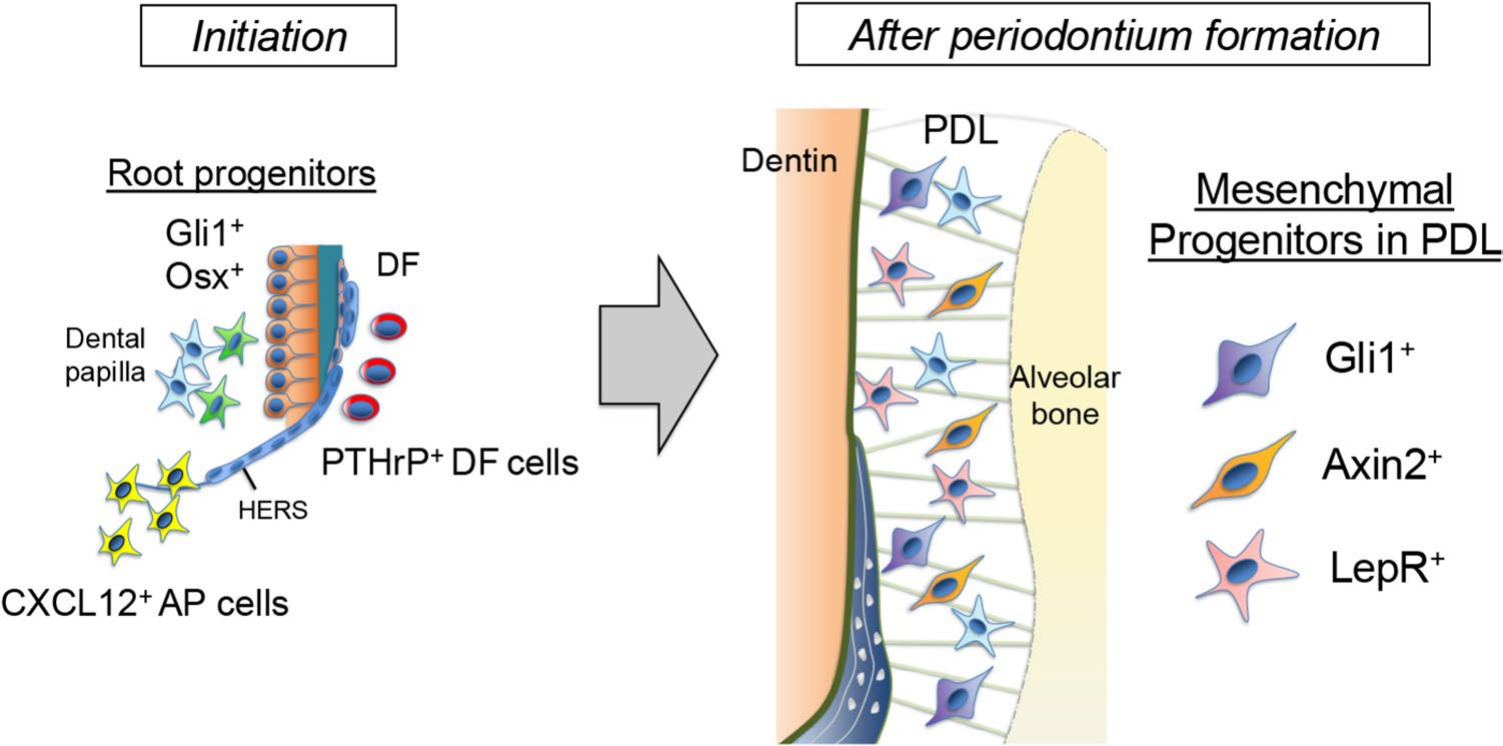
Developmental origin and stem-cell diversity of periodontium. Schematic diagrams of stem-cell populations in mice. During tooth root development, Gli1^+^ root progenitors are found in both the epithelial layer, such as HERS, and the mesenchyme, such as the apical papilla and the dental follicle. Osx^+^ root progenitors are found in the mesenchyme, such as the apical papilla and the dental follicle. CXCL12^+^ mesenchymal progenitors are specifically in the apical papilla. PTHrP^+^ mesenchymal progenitors are specifically in the dental follicle. After periodontium formation, Gli1^+^, Axin2^+^, and LepR^+^ mesenchymal progenitors in the PDL differentiate into PDL fibroblasts, cementoblasts and osteoblasts

Together, these findings support a model in which periodontal stem/progenitor cells are organized into a spatially defined and temporally regulated network, integrating niche-specific signals to coordinate tissue development, homeostasis, and regeneration (Fig. 2).

## Conclusions and perspectives

Recent advances in in vivo lineage-tracing establish the periodontium as a dynamic, stem-cell-regulated organ in which multiple mesenchymal progenitor populations derived from the DF, AP, and PDL cooperatively maintain cementum, PDL, and alveolar bone. Distinct progenitor lineages form a spatially organized and temporally regulated hierarchy governed by coordinated signaling pathways, including Hedgehog–Foxf, Wnt/β-catenin, BMP/TGF-β, and mechanotransduction. These insights revise classical models of periodontal development and regeneration by demonstrating that the attachment apparatus is maintained by heterogeneous, lineage-restricted progenitors rather than a uniform stem-cell pool.

A key conceptual advance is the recognition that effective periodontal regeneration relies on modulation of endogenous stem-cell niches rather than on exogenous cell transplantation. Spatial transcriptomic technologies represent a critical next step by enabling the simultaneous resolution of gene expression patterns [70–72]. Spatial transcriptomics is increasingly recognized as a critical tool for periodontal research, enabling cell-type-resolved analysis while preserving tissue architecture, and spatial profiling of gingival tissues has advanced rapidly [73–75]. However, comprehensive analysis of the entire periodontium remains limited, because mineralized tissues such as alveolar bone and cementum require decalcification, which compromises RNA integrity and spatial resolution.

Recent advances in spatial transcriptomic technologies may help overcome these limitations. Conventional platforms rely on the capture of polyadenylated mRNA [71] and are, therefore, highly susceptible to RNA degradation during the decalcification required for mineralized tissues. In contrast, probe-based and imaging-based approaches, such as MERFISH [76] and seqFISH [77], which detect short RNA fragments independently of poly(A) tails, demonstrate greater robustness in partially degraded or FFPE samples. These methods enable high resolution, spatially resolved transcriptomic analysis while preserving tissue architecture, offering a promising strategy for investigating hard–soft tissue interfaces, such as the alveolar bone–PDL–cementum complex. Developing spatial transcriptomic approaches compatible with hard–soft tissue interfaces will be essential for elucidating stem-cell niches and signaling gradients that govern periodontal regeneration.

Ultimately, understanding stem-cell regulation in the periodontium offers insights that extend beyond dental biology. The periodontium serves as a paradigm for load-bearing skeletal interfaces, where mechanical, inflammatory, and developmental cues intersect. The convergence of lineage tracing, single-cell and spatial transcriptomics, and bioengineering approaches promises to transform periodontal regeneration into a predictable and programmable biological process, with broad implications for regenerative strategies across craniofacial and musculoskeletal tissues.
